# Desmoplastic Small Round Cell Tumor of the Ovary: A Case Report with a New Modality of Treatment and Review of the Literature

**DOI:** 10.1055/s-0040-1710350

**Published:** 2020-05

**Authors:** Goran Vujić, Mislav Mikuš, Luka Matak, Aleksandra Bonevski, Ivan Babić, Pavao Planinić, Damir Babić, Ante Ćorušić

**Affiliations:** 1 Department of Obstetrics and Gynecology, University Hospital Center Zagreb, Zagreb, Croatia; 2 Department of Obstetrics and Gynecology, General Hospital Zadar, Zadar, Croatia; 3 Department of Oncology and Hematology, Children's Hospital Zagreb, Zagreb, Croatia

**Keywords:** desmoplastic small round cell tumor, ovary, surgical debulking, adjuvant chemotherapy, radiotherapy, immunohistochemistry

## Abstract

**Objective** Desmoplastic small round cell tumor (DSRCT) is a rare intraabdominal neoplasm that grows along serosal surfaces and is primarily found in young men. To date, only 16 cases of ovarian DSRCT have been previously reported in women in the English literature, and no large population-based studies on this topic exist.

**Case Report** We report the case of a 19-year-old virgo with unremarkable past medical history, initially presented with abdominal fullness. After being treated with the optimal treatment modality (primary and secondary surgical debulking, unique chemotherapy, protocol and adjuvant radiotherapy), the patient has remained without tumor disease for 40 months.

**Conclusion** Although the best therapy for patients with DSRCT has yet to be determined, combining complete surgical resection, adjuvant chemotherapy, and radiotherapy is required to prolong survival and to achieve proper quality of life.

## Introduction

Desmoplastic small round cell tumor (DSRCT) is a highly malignant, rare intraabdominal neoplasm of mesenchymal origin with an extremely poor prognosis. It primarily affects young men, with a reported male to female ratio of four to one.^1^ Desmoplastic small round cell tumor has a propensity for serosal surfaces, and the majority of the patients are in the late stages of the disease upon presentation.^2^ Owing to the male predominance of this disease, DSRCT is rarely noticed in young women with an abdominal or ovarian mass, so it presents a considerable diagnostic challenge. Furthermore, given the overall low incidence rate,^3^ uncertain histogenesis, and the diffuse nature of DSRCT, there is no consensus on the most effective treatment modality. Up to now, only 16 cases of ovarian DSRCT have been previously reported in women in the English literature, and no large population-based studies on this topid exist.^2^ The present report describes a case of DSRCT in a young woman who initially presented with right ovarian mass and consequent abdominal distension. The aim of the current review is to highlight all the issues encountered in diagnosing and treating the DSRCT.

## Case Presentation

A 19-year-old nulliparous virgo, with unremarkable past medical history, initially presented to our department with a feeling of abdominal fullness. Pelvic examination revealed a painless right adnexal mass that was occupying the whole abdominal cavity. The mass was firm, solid, of low mobility, and with hard-to-define boundaries. Transabdominal and transvaginal ultrasonography revealed a predominantly solid and vascularized, bilateral adnexal formation with an area of 80 mm. The level of the serum carbohydrate antigen-125 was elevated to 271 U/ml (normal, < 35 U/ml), while other serum tumor markers (β-HCG, AFP) were not elevated. The patient underwent an exploratory laparotomy. Approximately 1,000 ml of amber-colored cytologically negative ascites were withdrawn intraoperatively. Two large, irregular, and predominantly solid, bilateral ovarian masses (both ∼ 20 cm) were found with small tumor implants on the sigmoid colon, omentum, and cul-de-sac. The interiliac lymph nodes measured 4 cm, bilaterally. Using intraoperative frozen section diagnosis, the tumor tissue of right ovary was interpreted as a malignant tumor, probably dysgerminoma. A bilateral salpingo-oophorectomy, bilateral pelvic lymphadenectomy, total abdominal hysterectomy, complete omentectomy, and resection of the disseminated tumors with interiliac lymph node excision were performed. The patient was optimally debulked with no residual tumor seen. Macroscopically, the right and the left ovarian masses were pearly white, predominantly solid with partially pseudocystic areas and necrosis. Conventional hematoxylin and eosin stained sections demonstrated small and round tumor cell nests separated by a prominent desmoplastic stroma. The mitotic count of the tumor cells was high (up to 26 mitotic figures per 10 high power fields), suggesting a poorly-differentiated carcinoma. Immunohistochemical staining was performed using the antigen retrieval technique. The tumor cells were diffuse positive for broad-spectrum cytokeratins (CK AE1/AE3), epithelial membrane antigen (EMA), and vimentin. Polymerase chain reaction (PCR) analysis showed positive results for Ewing sarcoma (EWS)—Wilms tumor 1 (WT1) fusion gene, while additional confirmation of the DSRCT diagnosis was made via detection of the tumor-specific chromosomal translocation t(11;22)(p13;q12). Based on the morphology and immunohistochemistry findings, a final diagnosis of intraabdominal DSRCT with ovarian involvement was made. Bone marrow biopsy was performed after a final pathologic diagnosis and within three weeks after surgery, revealing normocellular pattern. Furthermore, a PET/CT scan demonstrated enlarged paraaortic lymph node in projection of third lumbar vertebrae with cluster of hyperdense nodes beside it, without metabolic activity. According to the CWS 2009 protocol scheme, she has received intensive chemotherapy treatment for metastatic disease: 3 cycles of ifosfamide 9,000 mg/m^2^, vincristine 1,5 mg/m^2^, actinomycin 1,5 mg/m^2^ (IVA), 3 cycles of carboplatin 500 mg/m^2^, epirubicin 150 mg/m^2^, Vincristine 1.5 mg/m^2^ (CEV), and 3 cycles of ifosfamide 9,000 mg/m^2^, vincristine 1,5 mg/m^2^, etopozide 450 mg/m^2^ (IVE) with informed consent of the patient and her family. Only in first cycle of IVA and in the first cycle of CEV she has received vincristine 3 × 1.5 mg/m^2^. Because of suspect malignancy propagation on positron emission tomography/computed tomography (PET/CT) scan, we conducted a second debulking surgery (SDS), about 4 months after initial surgical treatment. Intraoperative finding was a solid tumor (9 × 5 cm), barely fixed with external iliac vessels. We performed successful tumor resection with no residual tumor seen in the abdominal cavity. Cytological and immunohistochemistry findings confirmed diagnosis of DSRCT. The patient received nine cycles of CWS protocol overall. After the ninth cycle of CWS protocol, a disease remission was accomplished, and the patient underwent high-dose chemotherapy (busulfan 600 mg/m^2^, melphalan 140 mg/m^2^) followed by autologous stem cell support. After the autologous stem cell support, she received a couple of cycles of maintenance therapy (etoposide 2 × 25 mg/m^2^/day, idarubicin 4 × 5 mg/m^2^ and trofosfamide 2 × 75 mg/m^2^/day) but, due to drug induced high transaminase levels, we stopped it. The transaminase levels have normalised soon after. Twelve months after the patient's admission, a control PET/CT scan, pelvic and abdominal MRI were without evidence of eventual disease reccurence. In accordance with the above mentioned clinical findings, our approach was to provide a radiotherapy to the site of initial bulk disease (dose of 30.6 Gy divided in 17 fractions). The only noticeable adverse effect during the course of therapy was thrombocytopenia, which was extensive at one point, so it required treatment with intravenous immunoglobulins and platelet transfusion. Up to date, she is in good general condition without evidence of disease reccurence, 40 months after her initial diagnosis.

## Discussion

**Fig. 1:**
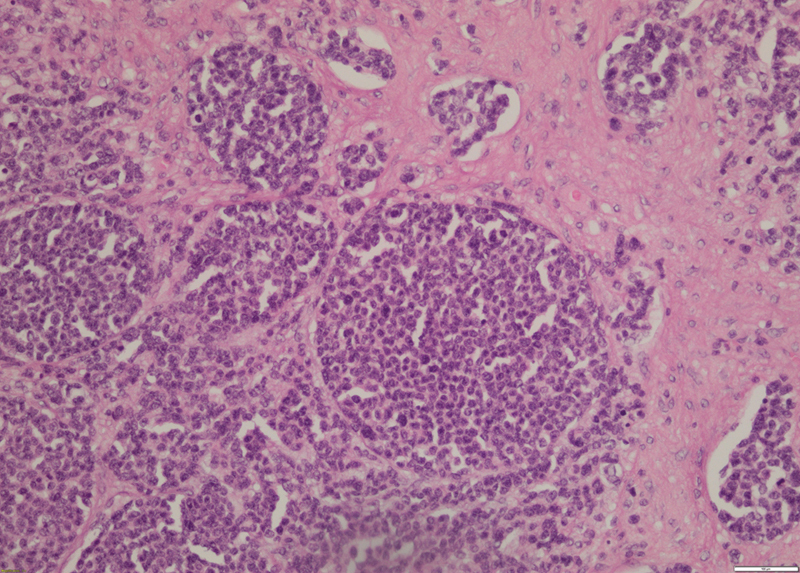
Microscopic finding of desmoplastic small round cell tumor, hematoxylin and eosin staining. **Source:** Engohan-Aloghe et al.^12^

Desmoplastic small round cell tumor was first described in 1989 by Gerald and Rosai^4^ as a rare tumor of uncertain histogenesis. The presenting symptoms of DSRCT are usually related to the site of involvement, such as crampy abdominal pain, abdominal distension with ascites, palpable mass, constipation, anorexia, or weight loss.^1^ ^2^ The most common primary location of DSRCT is the peritoneal cavity, but it can be found at other sites, such as the ovary, kidney, or retroperitoneal space.^5^ Primary DSRCT has also been reported in the posterior cranial fossa, scalp, ethmoidal sinuses, paratesticular region, pleura, and chest.^6^ ^7^ The typical sites of metastas isinclude the groin, neck or mediastinum, lymph nodes, liver, lungs, and bone marrow (5,10). Although the tumor markers in our case were within th reference values range, β-HCG, AFP, LDH, and CA-125 should be measured in all young women who present with a pelvic mass. Elevated serum tumor marker levels may serve as an adjunct in the initial diagnosis, therapy monitoring, and posttreatment surveillance. Conforming to tumor rarity and certain aggressive nature, it has low overall survival rates with reported 3-year survival of 29%,^8^ and median progression-free survival of 2.6 years.^9^ ^10^ The overall progression-free 5-year survival rate of patients is 18%.^11^ The mean age of affected women reported up to this point is 20 years with life expectancy range from 4 to 42 months (Table 1). Due to the rare occurrence of this disease, with up to 0.5 cases/million,^3^ a definitive diagnosis is only obtained based on pathological conclusion. Our microscopic findings indicated the DSRCT diagnosis (Fig. 1).^12^ An additional important and useful diagnostic tool is the combination of immunohistochemical staining and cytogenetic analysis. The characteristic DSRCT immunophenotype is the coexpression of epithelial, mesenchymal, and neuroendocrine markers.^13^ Furthermore, the small round cells in our material were focal positive for desmin and Wilms tumor 1 (WT-1) genes and were negative for chromogranin, among others, thus helping our pathologist to distinguish DSRCT from other forms of small round cell tumors (e.g., ovarian small cell carcinoma of hypercalcemic type). The expression of immunolabelings in the small round cells showed great level of variability (Table 2), with positive desmin and broad-spectrum CK being considered the most specific immunological indexes of DSRCT. If the diagnosis is uncertain following microscopic, immunohistochemical, or cytologic analysis, the PCR method or fluorescent in-situ hybridization (FISH) can be helpful in providing supplemental diagnostic information. Desmoplastic small round cell tumor exhibits a unique chromosomal transcription, resulting in the fusion of EWS-WT-1 to create a gene that presumably acts as a transcriptional activator for pro-tumorigenic genes.^14^ Because this chromosomal transcription is not always present (Table 2), the efficiency of this diagnostic approach, considering the eventual costs of the diagnostic tools used, is still controversial. Nevertheless, a lot of efforts are still required towards a definition of the most adequate therapeutic modality of choice. Although the multiple treatment strategies have been assessed, DSRCT survival has not significantly improved (Table 1). Currently, a combination of surgical resection and chemotherapy are commonly used for the initial treatment, although there is no consensus regarding whether surgical debulking should be preceded or followed by chemotherapy.^2^ In one reported study by Lal et al, there is a strong correlation between 3 and 5-year survival with multimodal therapy treatment. A systemic chemotherapy utilizing the P6 regimen, aggressive surgical debulking with greater than 90% surgical resection, and adjuvant radiotherapy combined demonstrated significant contribution to improved overall survival.^15^ In terms of choice of chemotherapy, DSRCT is alkylator-sensitive and dose-responsive, compared to other small round-cell tumors.^16^ Certain regimens, especially platinum-based ones, showed great overall safety profile and should be considered even in the second and early third trimester of pregnancy.^17^ Scheer et al^18^ demonstrated the best results with the vincristine, adriamycin, ifosfamide, actinomycin D (VAIA) scheme in a multivariable model proven by the Cox regression analysis as an independent effect. The median event-free survival (EFS) of 15 patients treated with VAIA was 29.4 months. Due to the poor prognosis and outcome for patients with DSCRT, we have decided to treat the young girl according to guidelines established by the Cooperative Weichteilsarkom Study (CWS) group in cooperation with the European pediatric SoftTissue Sarcoma Study Group (EpSSG) protocol scheme treatment plan for very high risk/metastatic patients, which we use at our department for the treatment of soft-tissue sarcomas. The CWS guildelines differ from the P6 protocol in number of cycles (P6 has 7 and CWS 9 cycles), and CWS lacks thecyclophospamide, but it has introduced the carboplatin and epirubicine to the first-line chemotherapy treatment (high-dose carboplatin has been used in the P6 protocol as a part of myeloablative regimen with stem-cell rescue). A possible connection with Ewing sarcoma was observed after we performed the PCR analysis and the EWS-WT1 fusion gene was revealed. Both tumors share an EWS fusion protein and may also share molecular mechanisms promoting proliferation and survival pathways. That explains why the vincristine, ifosfamide, doxorubicin, etoposide (VIDE) regimen was chosen to treat some cases, for which Wong et al^19^ reported a median time to progression of 14.6 months, and Frank et al^20^ reported survival of 19 months. In our report, the patient was treated with the CWS chemotherapy protocol scheme used for the treatment of rare mesenchymal tumors. The patient is still alive with EFS of 40 months. Furthermore, it is evident that adjuvant chemotherapy following gross total tumor resection is necessary to achieve long-term disease control in DSRCT, as shown in this particular case. Although the P6 regimen has been the cornerstone of initial DSRCT therapy, with great response in the majority of cases, it did not improve overall survival, suggesting an aggressive nature of the disease but also a requisite for another systemic, targeted treatment modality. There is no unique chemotherapy protocol in the treatment of DSRCT; thus, the choice is based on clinician experience or patient protocol indulgement. To our knowledge, our case is the first of all ovarian-involved DSRCT in the English literature to use CWS chemotherapy protocol (Table 1) with remarkable success. It is important to emphasize that one of the main difficulties in the treatment of DSRCT, due to its adhesive nature, is not achieving optimal surgical debulking, with more than 10% of tumor left.^1^ ^21^ ^22^ ^23^ ^24^ ^25^ ^26^ ^27^ We found optimal surgical debulking to be necessary for local disease control and for establishing better response on adjuvant treatment modalities. Other adjuvant treatment modalities may include hyperthermic intraperitoneal chemotherapy,^21^ ^22^ yttrium microsphere,^22^ or, as presented in our case, high-dose chemotherapy followed by autologous stem-cell support. We demonstrated the efficienty of the treatment modality chosen in this case, which combined primary and secondary surgical debulking, CWS chemotherapy protocol, and boost radiotherapy joined with high-dose chemotherapy followed by autologous stem cell support. The young woman from our case is still alive 40 months after the treatment, with great probability of being the patient with the longest survival period observed,^23^ since in her most recent follow-up appointment, in July of the present year, tno tumor disease was observed and she presented good overall physical and mental condition.

**Table 1:** Overview of the 16 reported cases of ovarian desmoplastic small round cell tumor

Case (n)	Reference	Age (yrs)	Ovarian involvement	Treatment modalities	Chemotherapy protocol	Follow-up
**1**	Young et al^24^	15	Unknown laterality	PDS + SDS + CHT	Multi-agent protocol including carboplatin	Succumbed at 4 months
**2**	Young et al^24^	15	Bilateral	PDS + SDS	Not used	Unknown
**3**	Young et al^24^	14	Right	PDS	Unknown, ifused	Unknown
**4**	Zaloudek et al^25^	22	Bilateral	PDS + CHT	BEP	Succumbed at 18 months
**5**	Slomovitz et al^26^	11	Right	PDS + CHT + ASCS	Modified P6 + myeloablative chemotherapy	Succumbed at 11 months
**6**	Parker et al^27^	23	Right	PDS + CHT	Platinum and taxol chemotherapy	Unknown
**7**	Elhajj et al^23^	27	Bilateral	PDS + CHT	Delayed C/E followed by CAV	Succumbed at 42 months
**8**	Ota et al^1^	26	Bilateral	PDS + CHT + SDS + RT	P6	Succumbed at 23 months
**9**	Ota et al^1^	19	Bilateral	PDS + CHT + SDS	BEP	Succumbed at 11 months
**10**	Fang et al^14^	13	Left	PDS + CHT + RT	BEP	Succumbed at 21 months
**11**	Fang et al^14^	23	Bilateral	PDS + CHT	Myeloablative chemotherapy	Alive at 7 months
**12**	Engohan-Aloghe et al^12^	21	Bilateral	PDS + CHT	unknown	Alive at 7 months
**13**	Nakayama et al^28^	6	Bilateral	PDS + CHT + RT + CHT	P6 + IMC-A12 trial + temsirolimus	Succumbed at 28 months
**14**	Nakayama et al^28^	28	Right	CHT + SDS	Neoadjuvant P6 (with the removal of adriamycin) + topotecan after surgery	Succumbed at 40 months
**15**	Nakayama et al^28^	17	Right	PDS + CHT + SDS + RT	P6	Alive at 11 months
**16**	Xie and Shen^2^	30	Right	PDS + CHT	VAC	Alive at 15 months
**17**	Present case	19	Bilateral	PDS + CHT + SDS + RT + ASCS	CWS + Bu-Mel	Alive at 40months

Abbreviations: ASCS, autologous stem cell support; BEP, bleomycin, etoposide and cisplatin; Bu-Mel, busulfan, melphalan; C/E, cisplatin/etoposide; CHT, chemotherapy; CWS, etoposide, idarubicin, and trofosfamide; PDS, primary debulking surgery; RT, radiotherapy; SDS, secondary debulking surgery; VAC, vincristine, adriamycin, and cyclophosphamide.

**Source:** Modified from Xie and Shen.^2^

**Table 2:** Result of immunohistochemical studies—expression of immune labelings in part of reported ovarian desmoplastic small round cell tumor cases

Reference	Des	AE1/AE3	CK7	Inh	Vim	EMA	NSE	Chr	Actin	WT1	EWS/WT1 (PCR)
1	+ +	+ +	−	N/A	N/A	+ +	N/A	−	−	+	NP
1	+ +	+ +	+	N/A	N/A	+ +	N/A	−	−	+	P
2	+ + (*)	+ +	N/A	−	+ +	+ +	−	N/A	N/A	N/A	N/A
12	+ + (*)	−	N/A	+	N/A	−	+ +	−	+	N/A	P
18	+	N/A	N/A	N/A	+	N/A	+	N/A	+	N/A	N/A
19	+	N/A	N/A	N/A	+	+	+	−	−	N/A	N/A
19	+	+ +	N/A	N/A	+ +	+ +	−	−	+ +	N/A	N/A
19	+	+ +	N/A	N/A	+	+ +	+ +	−	−	N/A	N/A
21	+	+	+	N/A	N/A	N/A	N/A	N/A	N/A	N/A	N/A
23	+	N/A	N/A	N/A	N/A	+	+	N/A	N/A	N/A	P
23	+ +	+ +	+ +	N/A	N/A	N/A	N/A	N/A	N/A	N/A	NP
23	+ +	+ +	+ +	−	N/A	N/A	N/A	N/A	N/A	−	P
Present case	+	+ +	−	+	+ +	+ +	−	−	−	+	P

Abbreviations: (-), negative; (*), dot-likepattern; (+ +), diffuse positive; (+), focally positive; AE1/AE3; CK7, cytokeratin markers; Chr, chromogranin; Des, desmin; EMA, epithelial membrane antigen; EWS/WT1, Ewins sarcoma/Wilms tumor 1; Inh, inhibin; N/A, information not available or test was not done; NP, not present; NSE, neuron-specificenolase; P, present; PCR, polymerase chain reaction; Vim, vimentin.

## Conclusion

Desmoplastic small round cell tumor is an unusual and remarkably malignant tumor that affects the young population, with a small number of long-term survivals. An increasing number of patients has been diagnosed with DSRCT over past decade, and, with improvement of our overall knowledge, especially about the molecular mechanisms and signal pathways involved in the tumor pathogenesis, we can expect further advances in treatment. Although the best therapy for patients with DSRCT has yet to be determined, combining complete surgical resection, adjuvant chemotherapy, and radiotherapy is required to prolong survival and to achieve proper quality of life.
